# Combination of Girdlestone Pseudoarthroplasty and Negative Pressure Wound Therapy with Instillation and Dwell in the Treatment of Invasive Osteomyelitis of the Proximal Femur

**DOI:** 10.7759/cureus.3552

**Published:** 2018-11-06

**Authors:** Nirbhay Jain, Christopher B Horn, Erin G Andrade, Laurie Punch

**Affiliations:** 1 Surgery, Washington University, St. Louis, USA; 2 Surgery, St. Louis University, St. Louis, USA; 3 Surgery, Washington University, Barnes-Jewish Hospital, St. Louis, USA

**Keywords:** wound instillation, chronic suppurative osteomyelitis, femur, bone infection, pressure ulcers, osteomyelitis, negative pressure wound therapy, girdlestone, pressure ulcer, paraplegia

## Abstract

Osteomyelitis is a progressively destructive invasive infection of the bone that can result in both localized and systemic illness. This includes an acute suppurative infection, generalized weakness, a failure to thrive, a pathological fracture, and non-healing ulcers. When chronic osteomyelitis develops, therapeutic options are limited, as antimicrobial agents cannot penetrate the necrotic bone, and repeated surgical debridement may be needed. Re-establishing full thickness coverage of the wounds and ulcers associated with osteomyelitis is challenging due to factors such as ongoing pressure injury, malnutrition, and resistant microorganisms. Classically, Girdlestone pseudoarthroplasty has been used to manage a resistant and invasive infection of the acetabular cavity and proximal femur, but it is now rarely employed because of the morbidity of removing the femoral head and leaving a wound to heal by secondary intention. Negative pressure wound therapy with instillation and dwell (NPWTi-d) offers a powerful adjunct to the management of complex infections and wound healing. In this case series of invasive osteomyelitis of the proximal femur in non-ambulatory patients, we demonstrate that the combination of the Girdlestone and negative pressure wound therapy with instillation and dwell allows for delayed closure within a week of the initial procedure, with favorable outcomes and no recurrence of osteomyelitis.

The case log of a single surgeon was analyzed retrospectively over an 18-month period. The case series includes all patients who underwent the Girdlestone procedure for invasive osteomyelitis of the femoral head after failed antibiotic management, were non-ambulatory, and were greater than age 18.

A total of 10 patients with 11 Girdlestone operations were found. Patients were predominantly male. The average age was 40 years. All patients were treated with NPWTi-d and then underwent a delayed primary or partial closure on an average of 4.5 days after the initial debridement. All four patients with no pre-existing pressure ulceration of the greater trochanter underwent primary closure without wound complication. Of the remaining patients with pre-operative ulcers of the greater trochanter, three were closed successfully or completely healed secondarily and four had substantial wound healing and reduction in size in the post-operative time period. All but one patient who had pre-operative ulcers of the ipsilateral ischium also had a noted improvement of ulcer dimensions in the postoperative follow-up period. Two patients developed new pressure ulcers on the contralateral side and two patients had a worsening of their pre-existing contralateral pressure ulcers more than 30 days post-operatively. No patient had a recurrence of their osteomyelitis. During the same time period, one patient refused surgical intervention and died secondary to overwhelming sepsis.

Girdlestone pseudoarthroplasty is a radical therapy for refractory invasive osteomyelitis. While it has been historically associated with prolonged or failed wound healing, combining this surgery with negative pressure wound therapy with instillation and dwell allows for the successful eradication of infection. In addition, this facilitates wound healing and closure, providing a powerful alternative to the challenge of refractory invasive osteomyelitis of the hip, an ultimately life-threatening infection.

## Introduction

Osteomyelitis is an infection of the bones that can result in the loss of the periosteum and cortex [1-2]. In the acute setting, this process has multiple etiologies, including local bacterial invasion from abdominopelvic sources, pressure ulceration, open fractures, and hematogenous spread to the bone, though the latter is rare in adults. Failure to eliminate the acute infection can lead to the development of a chronic osteomyelitis and inflammatory response that can result in pathological fracture, soft tissue ulceration, and acute suppurative infection. In patients with a spinal cord injury, the incidence of pressure ulcers is as high as 25%-66%, which makes this population especially high risk for the development of chronic osteomyelitis [3]. Stage IV ulceration of the ischium and greater trochanter, with superficial osteomyelitis, can give rise to severe invasive osteomyelitis with a subsequent infection of the acetabulum that can spread to the joint space of the hip. This infectious process is not well treated by systemic antimicrobial agents for many reasons, including poor penetration beyond the cortex of the bone as well as a sequestration of infected fluid within the joint space itself, resulting in septic arthritis [4]. Often, patients require surgical debridement for source control of the infection. With source control, a foundational principle in infectious disease, previously ineffective antimicrobial therapy can better penetrate the infected space and eliminate pathogens, improving therapeutic success rates [5-7]. However, in the most difficult cases, patients undergo multiple rounds of debridement and antimicrobials, including flap reconstruction, without establishing source control, which allows the infection to persist.

A classic approach to surgical infections of the acetabular cavity and femoral head is Girdlestone pseudoarthroplasty, which accesses the acetabulum, removes the femoral head and neck, and lets the wound heal by secondary intention to help drain purulent material [8-10]. However, Girdlestone pseudoarthroplasty has fallen out of favor, as the removal of the femoral head impairs hip function and advanced antimicrobial treatment improves outcomes without sacrificing ambulation. Nevertheless, the Girdlestone procedure still has a role in improving the quality of life in non-ambulatory patients with chronic osteomyelitis.

NPWTi-d for highly infected wounds has been shown to be associated with improved wound care. Specifically, the instillation and dwell component intermittently flushes and cleanses wounds, limiting bacterial burden and biofilm deposition and augmenting antimicrobial action [11-15]. Combining this with negative pressure into NPWTi-d increases local blood flow, promotes oxygenation, drains fluid, and stimulates inflammatory cells to improve pathogen clearance and the deposition of granulation tissue. This promotes granulation tissue deposition during the wound healing process, improving healing times and promoting wound closure. Though NPWTi-d is contraindicated in untreated acute osteomyelitis [16], it has a role in the management of treated infections.

In this study, we demonstrate a series of cases of invasive osteomyelitis in the femoral head and acetabulum treated with the Girdlestone procedure and NPWTi-d with a subsequent resolution of all infection.

## Case presentation

Methods

**Figure 1:**
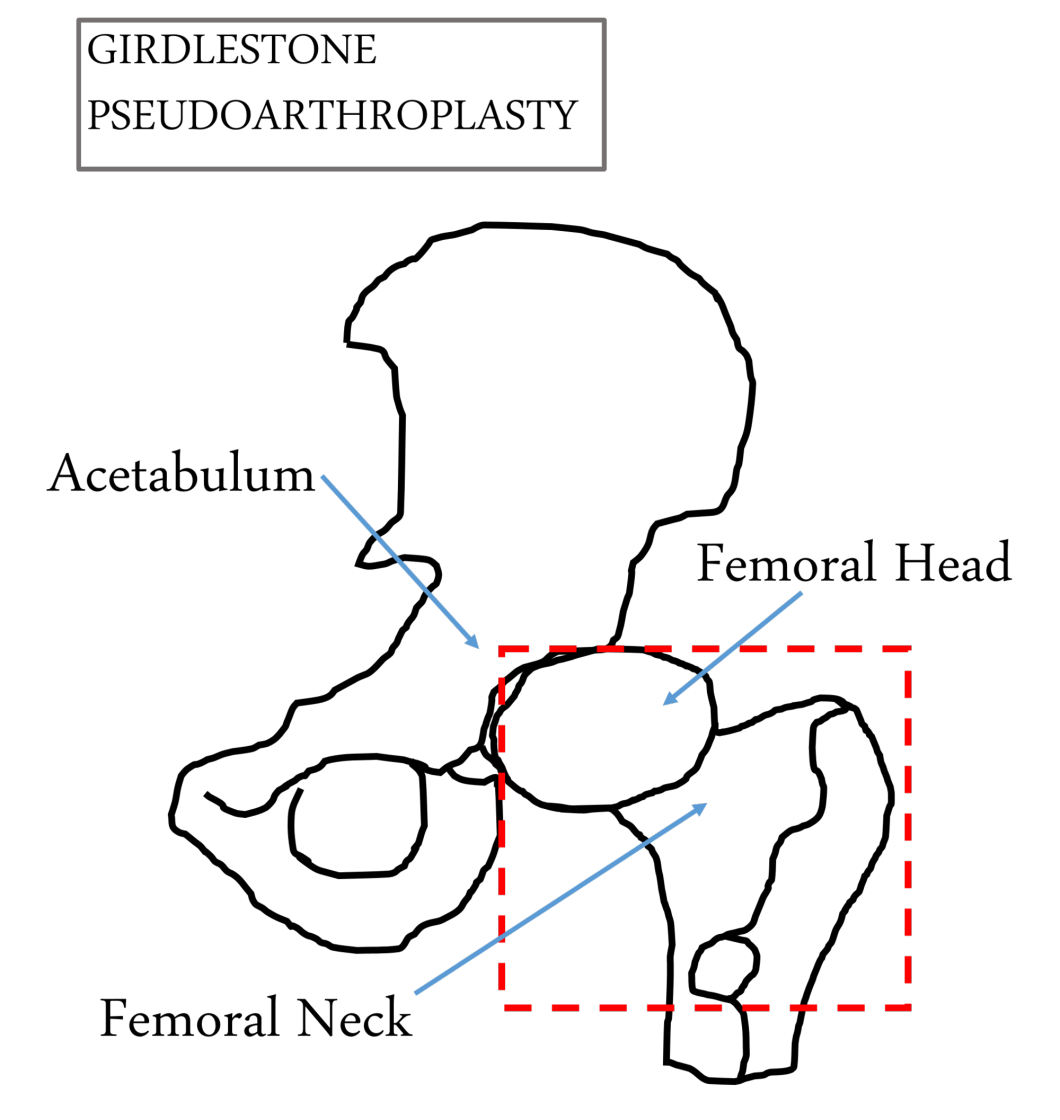
Resected Portion of Proximal Femur During Girdlestone Pseudoarthroplasty

For this retrospective series, the case log of a single surgeon from a large academic health center was reviewed from November 2016 to May 2018. Inclusion criteria specific to this study were: undergoing the Girdlestone procedure for an indication of invasive osteomyelitis of the acetabulum and femoral head or septic arthritis of the hip, failure of antibiotic therapy to control the infection, non-ambulatory, minimum one-month follow-up, and age > 18. Negative pressure wound therapy alone or NPWTi-d was utilized in all cases as interval and final wound care, along with incisional therapy with V.A.C. Ulta® (KCI, San Antonio, Texas, USA), as clinically indicated. Our standard NPWTi-d protocol, instillation with normal saline for a dwell time of 10 minutes followed by negative pressure wound therapy for 3.5 hours was followed unless otherwise indicated. Invasive osteomyelitis was defined as progressive bony destruction of the femoral head and/or acetabulum, suppurative infection of the hip, and associated systemic signs of illness. Figure 1 shows the classic approach of Girdlestone pseudoarthroplasty.

Operative information was analyzed, with preoperative indications, antibiotics, intraoperative wound measurements, and postoperative plans studied. If available, specifics on the amount of instillation fluid were discussed. Postoperative follow-up visits were reviewed for a minimum of one month. If postoperative follow-up evaluations past one month were available, those were also reviewed. All patients were assessed by the infectious disease team as well and treated with a culture-driven course of intravenous antibiotics for at least six weeks.

Results

Our review of the case log resulted in 10 patients with 11 Girdlestone procedures (nine unilateral, one bilateral) within our two-year study period. Patients were predominantly male (90%). The average age was 40 years. Operative cultures were polymicrobial in 10/11 cases. Methicillin-resistant Staphylococcus aureus (MRSA) was the most common pathogen, present in six of 11 cases. Delayed partial and primary closure occurred an average of 4.5 days (median day three) after the initial debridement (see Table 1). In the four patients without preoperative greater trochanter ulcers who underwent primary closure over a drain with topical negative pressure therapy after closure for five to seven days, there were no local wound complications. In the remaining five patients with pre-existing ulcers, two underwent complete primary closure while the rest underwent partial closure. After final closure, there were no surgical site infections nor post-operative hemorrhage. Complications included wound dehiscence in one patient and further dislocation of the femur in another. Two patients developed new pressure ulcers of the ischium and greater trochanter on the contralateral side from their procedure. Three other patients had a progression of a pre-existing ulcer on the contralateral side, with one undergoing a Girdlestone procedure for that ulcer and the other being evaluated for such a surgery. No patients were re-admitted within 30 days.

**Table 1:** Patient Characteristics * refers to presence of greater trochanter ulcer on the operative side Pt= patient DPC= delayed primary closure HTN= hypertension DM= diabetes mellitus MRSA= Methicillin resistant staph aureus PVD= peripheral vascular disease CAD= coronary artery disease

Pt #	Age	Sex	Operative Side	Comorbidities	Greater Trochanter Ulcer*	Culture Growth	Time to DPC	Closure
1	27	M	L	Paraplegia, HTN, tobacco use	No	S. aureus, B. fragilis	5	Complete
2	45	M	L	Quadriplegia, Type 1 DM	No	MRSA	5	Complete
3	70	M	R	Paraplegia	Yes	P. aeruginosa, Cladophialophora	4	Partial
4	35	M	R	Paraplegia	No	P. mirabilis	3	Complete
5	40	F	R	Quadriplegia	Yes	P. aeruginosa, E. coli, E. faecalis, MRSA, A. baumanni	3	Partial
6	25	M	L	Spina bifida	Yes	MRSA, P. mirabilis	3	Partial
7	29	M	R	Paraplegia	Yes	MRSA, S. epidermidis	3	Partial
7			L	Paraplegia	Yes	P. aeruginosa	3	Partial
8	59	M	R	Paraplegia, tobacco use	Yes	S. capitis, Candida, A. baumanni	4	Complete
9	52	M	L	Paraplegia, PVD, CAD, HTN, tobacco use	Yes	C. striatum, P. bivia, S. epidermidis, A. odontolyticus, E. faecalis, MRSA	3	Complete
10	21	M	R	Paraplegia, tobacco use	No	MRSA, E. coli, B. fragilis	13	Complete

Overall, this study shows that Girdlestone pseudoarthroplasty, when combined with instillation and dwell therapy, is an effective procedure in controlling resistant osteomyelitis of the femoral head in non-ambulatory patients. In addition, this demonstrates the effectiveness of utilizing a multi-modal approach to wound care, incision management, offloading, nutrition, and anti-microbial treatment to achieve wound closure and healing in the setting of stage IV pressure ulceration and invasive osteomyelitis of the hip.

Patient 1

This was a 27-year-old male with known paraplegia and chronic osteomyelitis who presented with stage IV pressure ulcers of his sacrum and left ischium. Despite previous antibiotic therapy, he developed invasive osteomyelitis of his left femoral head and underwent a Girdlestone procedure for further care. Intraoperative findings included a necrotic femoral head as well as areas of abscess and necrotic tissue. Cultures showed Bacteroides fragilis and Staphylococcus aureus. After the completion of the Girdlestone procedure, he had NPWTi-d placed in his surgical wound with 40 milliliters (mL) of normal saline following our standard NPWTi-d protocol using Veraflo® (KCI, San Antonio, Texas, USA). Five days after the initial procedure, he underwent delayed primary closure over closed suction drains with the placement of an incisional negative pressure device. His treatment while hospitalized included dedicated offloading bedding, nutrition supplementation, and culture-driven intravenous antimicrobial medications. He was then discharged on ciprofloxacin, vancomycin, and metronidazole antibiotic therapy seven days after the initial procedure. He had no readmissions in the first 30 days after discharge.

He was discharged two days after his delayed primary closure and followed up in clinic one week after the closure. His wound was healing well. His negative pressure device was removed at that time. One month after the operation, his sutures and staples were removed due to no sign of a secondary breakdown of the wound. He was then discharged to care at his local wound clinic for the management of his pressure ulcers with no recurrence of invasive osteomyelitis of the left hip.

Patient 2

This patient was a 45-year-old male with known quadriplegia and stage IV pressure ulcers of the ischium bilaterally, who presented with chronic osteomyelitis in his left femoral head and chronic septic arthritis due to MRSA in his acetabular space. As his infection was resistant to intravenous antibiotics, he underwent a left Girdlestone procedure. Intraoperative findings were significant for areas of inflammation in the greater trochanter. Cultures were negative. The surgical wound was 15 cm x 5 cm x 10 cm. His wound was dressed with Veraflo NPWTi-d. Five days later, he underwent completion debridement and delayed primary closure over closed suction drains with Prevena® (KCI, San Antonio, Texas, USA). He was discharged on vancomycin and meropenem four days after the initial procedure. Postoperatively, his negative pressure dressing was removed at his follow-up appointment five days after discharge and six days after closure. He continued to have no sign of a wound breakdown over the Girdlestone at the one-year follow-up. While his left ischial ulcer healed significantly, with no recurrence of infection in his left hip, he developed a worsening of his right ischial pressure ulcer two months postoperatively. He has not had a recurrence of his osteomyelitis or septic arthritis.

Patient 3

**Figure 2:**
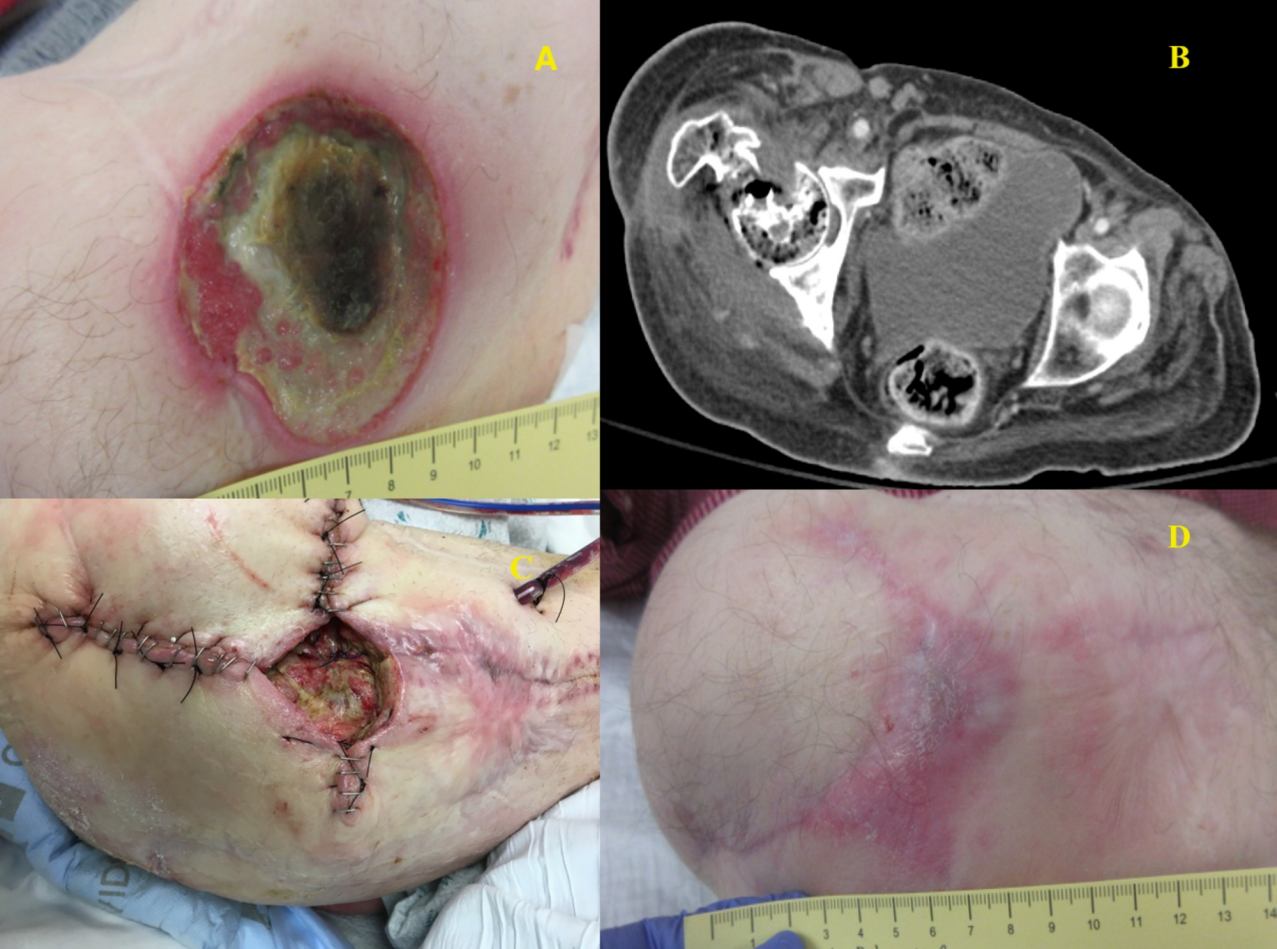
Patient 3 A: Greater trochanter ulcer; B: Pre-operative CT scan; C: Partial closure; D: Healed wound

This was a 70-year-old male with a history of paraplegia, a stage IV pressure ulcer of the right ischium, and new onset ulceration of the right greater trochanter in the setting of prior flap coverage and internal plating for prior fracture and pressure ulceration that had since healed well. Despite appropriate wound care, offloading, antimicrobial treatment, and removal of the hardware, the patient progressed to invasive osteomyelitis of his femoral head. The patient presented in sepsis and was admitted for a right-sided Girdlestone procedure. Intraoperative findings revealed a grossly necrotic bone with drainage of the cavity and cultures grew Cladophialophora mold, with concomitant sacral and ischial ulcers growing Pseudomonas. He was dressed with a Cleanse® (KCI, San Antonio, Texas, USA) negative pressure dressing with instillation and dwell with 50 mL of normal saline fluid instilled using our standard protocol. The wound was 10 cm x 11 cm x 5 cm and, therefore, not amenable to complete primary closure. Thus, he underwent delayed partial closure over closed suction drains four days later, with a negative pressure device over the incision and wound. He was discharged on six weeks of ertapenem and additionally had fluconazole for 10 days after the initial procedure. He had no readmissions in the first 30 days after discharge. His wound completely healed at 4.5 months postoperatively, with no subsequent infection of the treated hip one year after surgery. Figure 2 below demonstrates the preoperative ulcer of the greater trochanter with necrosis, computed tomography (CT) imaging of the fractured right femoral neck, postoperative wound closure, and the final healed wound.

Patient 4

**Figure 3:**
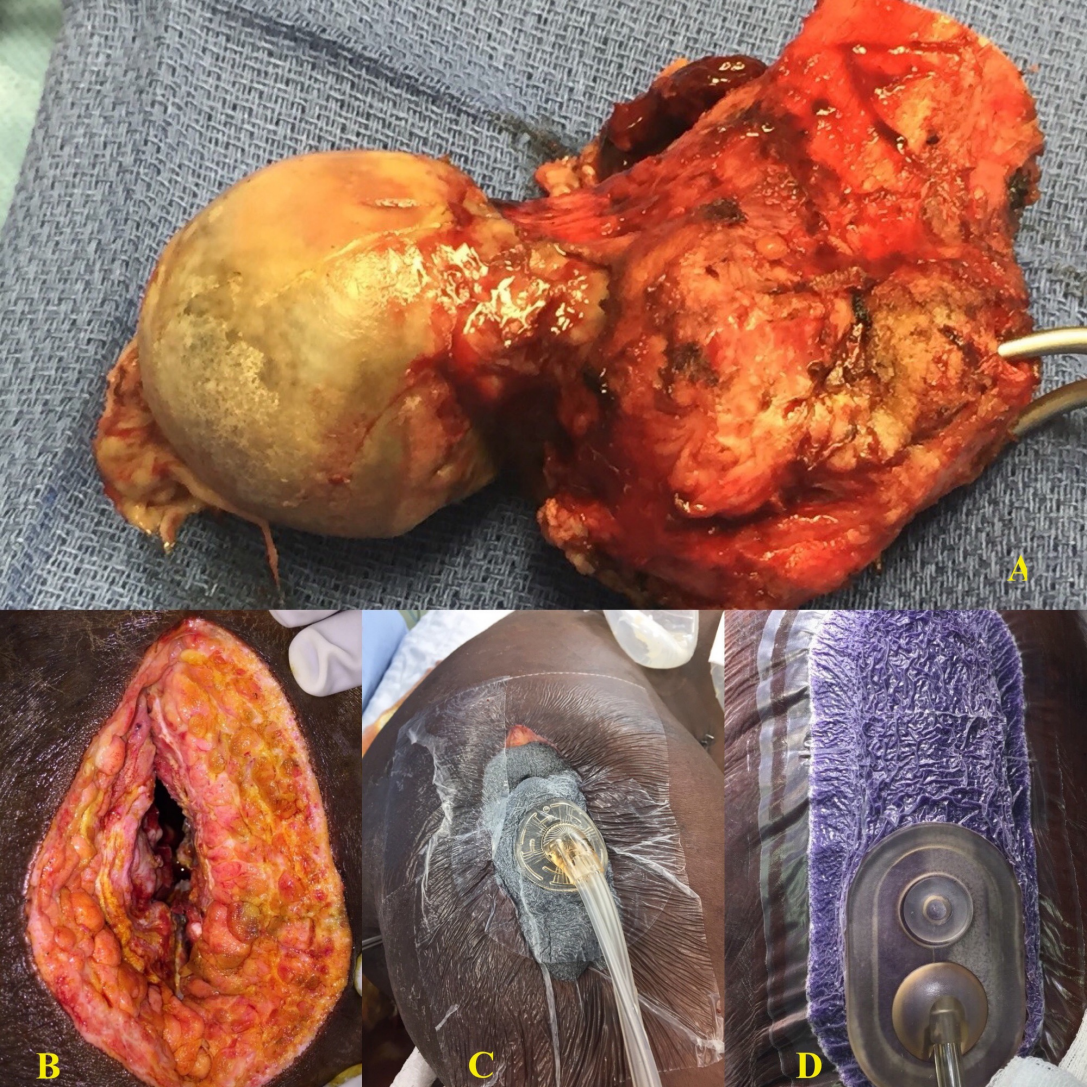
Patient 4 A: Resected femoral head; B: Post-operative wound; C: Application of instillation therapy; D: Closed wound with incisional vac (vacuum-assisted closure)

This patient was a 35-year-old paraplegic male who had a history of bilateral stage IV pressure ulcers of the ischium and presented with a disseminated Proteus infection involving his chest wall, right iliacus muscle, and right hip with associated osteomyelitis of several right-sided ribs, the acetabulum, and the femoral head. After initial stabilization with drainage of his iliacus and chest wall abscess along with nutritional supplementation, he underwent a Girdlestone procedure on his right hip. Intraoperative findings were significant for copious purulence and a grossly necrotic femoral head and a soft tissue capsule that spread to the acetabulum. These tissue cultures also grew Proteus species. His wound was dressed with a Cleanse NPWTi-d with a 50 mL lavage of ¼ strength Dakin's solution for a 10-minute dwell time every 3.5 hours. He underwent delayed primary closure over closed suction drains three days later and the incision was dressed with a Prevena. He had no readmissions in the first 30 days after discharge, no recurrent infections, and no wound complications. At the one-year follow-up, he presented with a significant reduction in the size of both ischial ulcers. Figure 3 below demonstrates his resected femoral head, the resultant wound after Girdlestone, the placement of instillation therapy, and final closure.

Patient 5

This was a 40-year-old quadriplegic female with known bilateral hip dislocations and multiple decubitus ulcers. She developed chronic septic arthritis in the right acetabulum with concurrent osteomyelitis, which progressed to an ulceration of the femoral head externally through her stage IV ischial pressure ulcer. Due to this ulceration and the risk of further ulceration on the right side, she underwent a Girdlestone procedure. Intraoperatively, her femoral head and neck were grossly necrotic, although surrounding soft tissue appeared to be healthy. Intraoperative cultures grew Pseudomonas, Escherichia coli, Enterococcus faecalis, MRSA, and Acinetobacter calcoaceticus-baumannii complex. Her resulting wound bed, including the acetabulum, was dressed with a Cleanse NPWTi-d utilizing normal saline. Three days later, she underwent partial delayed primary closure over closed suction drains with the placement of a negative pressure device over the incision and an ongoing open wound, as complete closure over the ischial ulceration was not possible. She was discharged 13 days after the initial procedure on ampicillin-sulbactam, vancomycin, and cefepime. She was not readmitted in the first 30 days after discharge.

She underwent a superficial debridement of her ongoing right ischial pressure ulcer at the one-month follow-up with healthy tissue found underneath. Due to the development of a new persistent left ischial and greater trochanter ulcer, she is being evaluated for a left-sided Girdlestone.

Patient 6

This was a 25-year-old male with known spina bifida who presented with a chronic infection of his left acetabulum. He had been previously managed for several years for a non-healing pressure ulcer of the left greater trochanter, having undergone a partial femoral head resection and prior flap placement with subsequent failure. He presented with large volume drainage from a small ulceration over his left trochanter with CT imaging demonstrating an abscess in the gluteus muscle with osteomyelitis in the abutting femoral head. He underwent a left Girdlestone procedure. Intraoperative findings included heterotopic ossification with necrotic bone in the femoral head. Cultures grew MRSA, Proteus mirabilis, and mixed microorganisms. The surgical wound was treated with a Cleanse NPWTi-d utilizing normal saline. Three days later, he underwent partial delayed primary closure over closed suction drains with the placement of a negative pressure dressing over the incision and ongoing wound, as complete primary closure was not possible due to the dimensions of the resulting wound. He was discharged eight days after the initial procedure on ertapenem. He was not readmitted in the first 30 days after discharge.

**Figure 4:**
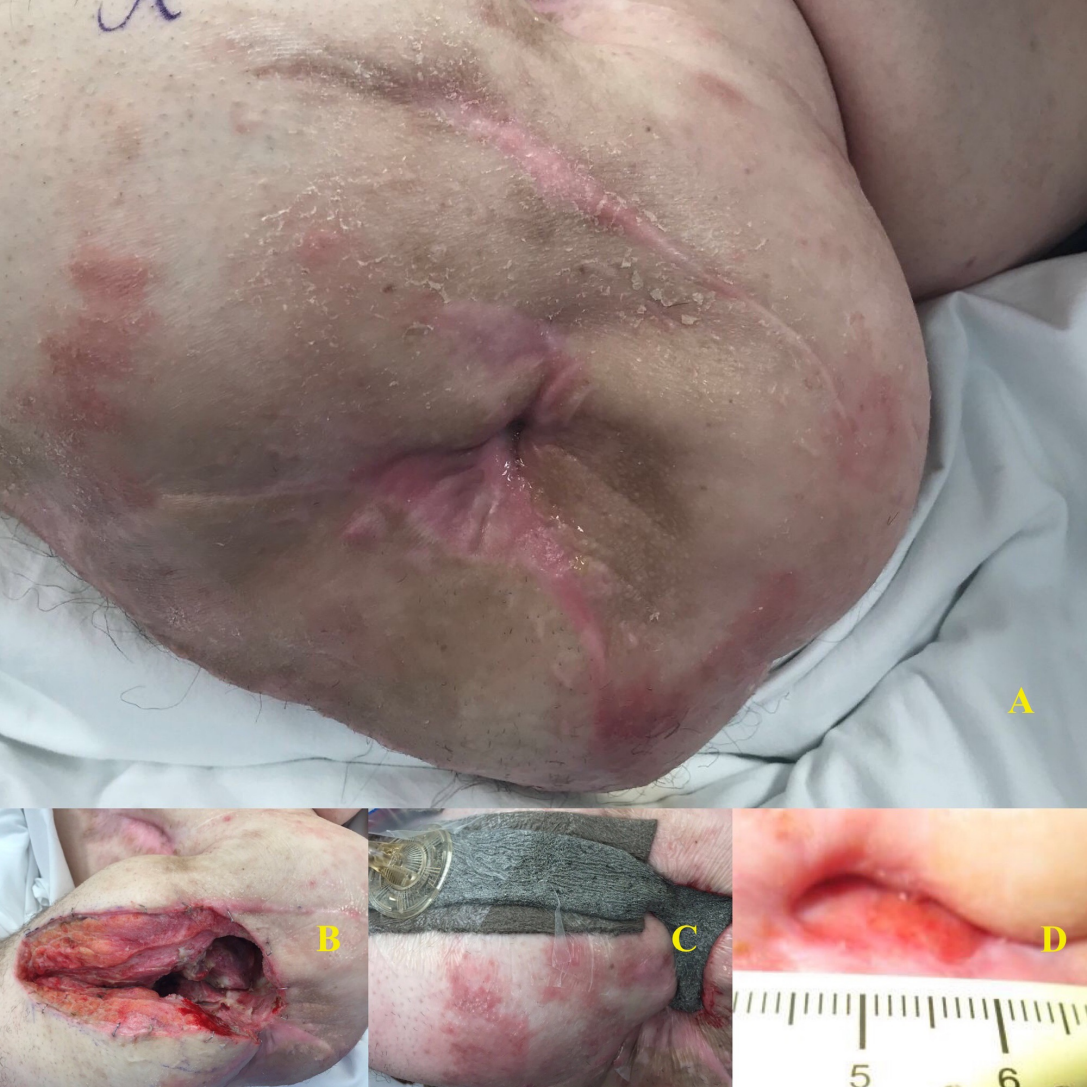
Patient 6 A: Chronic, draining trochanteric ulcer; B: Post-operative wound; C: Negative pressure wound therapy over closed incision; D: Healed wound with 1.5 cm ulceration

At his one-month follow-up, it was noted that his left-sided osteomyelitis had not recurred nor progressed. At his two-month visit, the wound continued to be clean and closed, with no sign of breakdown. However, at this time, he developed the worsening of a previously existing stage IV right ischial pressure ulcer, which was treated with operative debridement. He has not had a recurrence of his left hip osteomyelitis and his wound is nearly completely healed. Figure 4 below depicts his chronic trochanteric ulcer, the wound after Girdlestone resection, placement of negative pressure wound therapy over the closed incision, and the resultant healing wound.

Patient 7: right side

This was a 29-year-old male with a history of paraplegia who developed several stage IV ischial and sacral pressure ulcers on his right side, resulting in a dislocation of his femoral head on the right and progression of the infection into the acetabulum and iliacus muscle. His ulcer progressed despite appropriate treatment, and he also developed severe protein malnutrition; he was thus treated with a right Girdlestone procedure. Intraoperative findings were significant for necrotic exposed acetabulum and femoral head. Cultures grew MRSA and Staphylococcus epidermidis. The resulting wound bed, including the acetabulum, was dressed with a Cleanse Choice® (KCI, San Antonio, Texas, USA) NPWTi-d utilizing normal saline. Three days later, he underwent a partial delayed primary closure over closed suction drains with the placement of a negative pressure device over the incision. He was discharged 14 days after the initial procedure on doxycycline and trimethoprim-sulfamethoxazole. He was not readmitted in the first 30 days after discharge.

**Figure 5:**
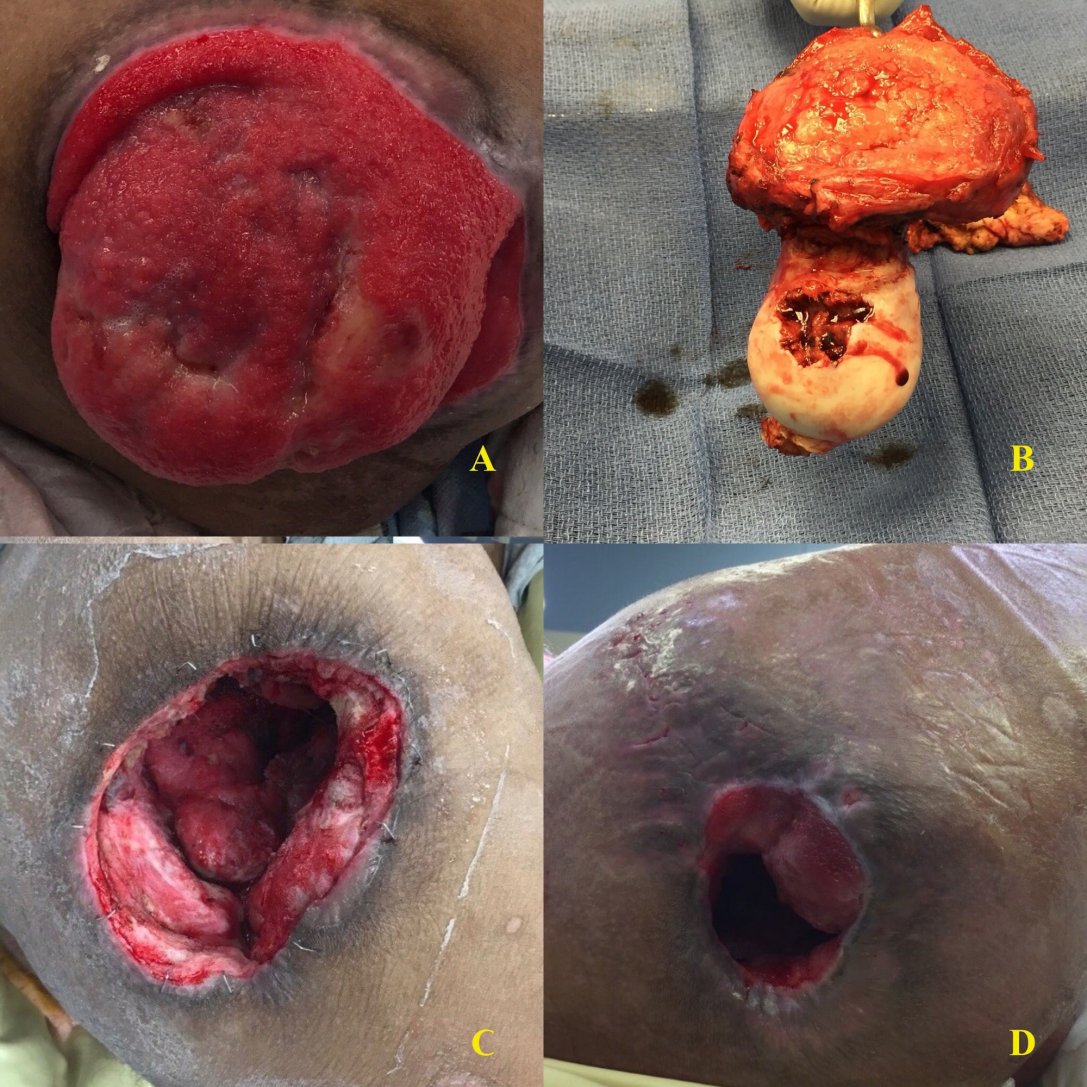
Patient 7: Right Hip A: Pre-operative ulcer; B: Resected femoral head; C: Wound after instillation therapy; D: Healing wound

At his three-month follow-up visit, his wound was healing well, with no sign of recurrent osteomyelitis on the right side. However, he did have progressive ulceration of his previously existing left greater trochanter ulcer and was found to have invasive osteomyelitis in the left hip. Figure 5 demonstrates the pre-operative ulcer, resection specimen, and resultant healing wound.

Patient 7: left side

**Figure 6:**
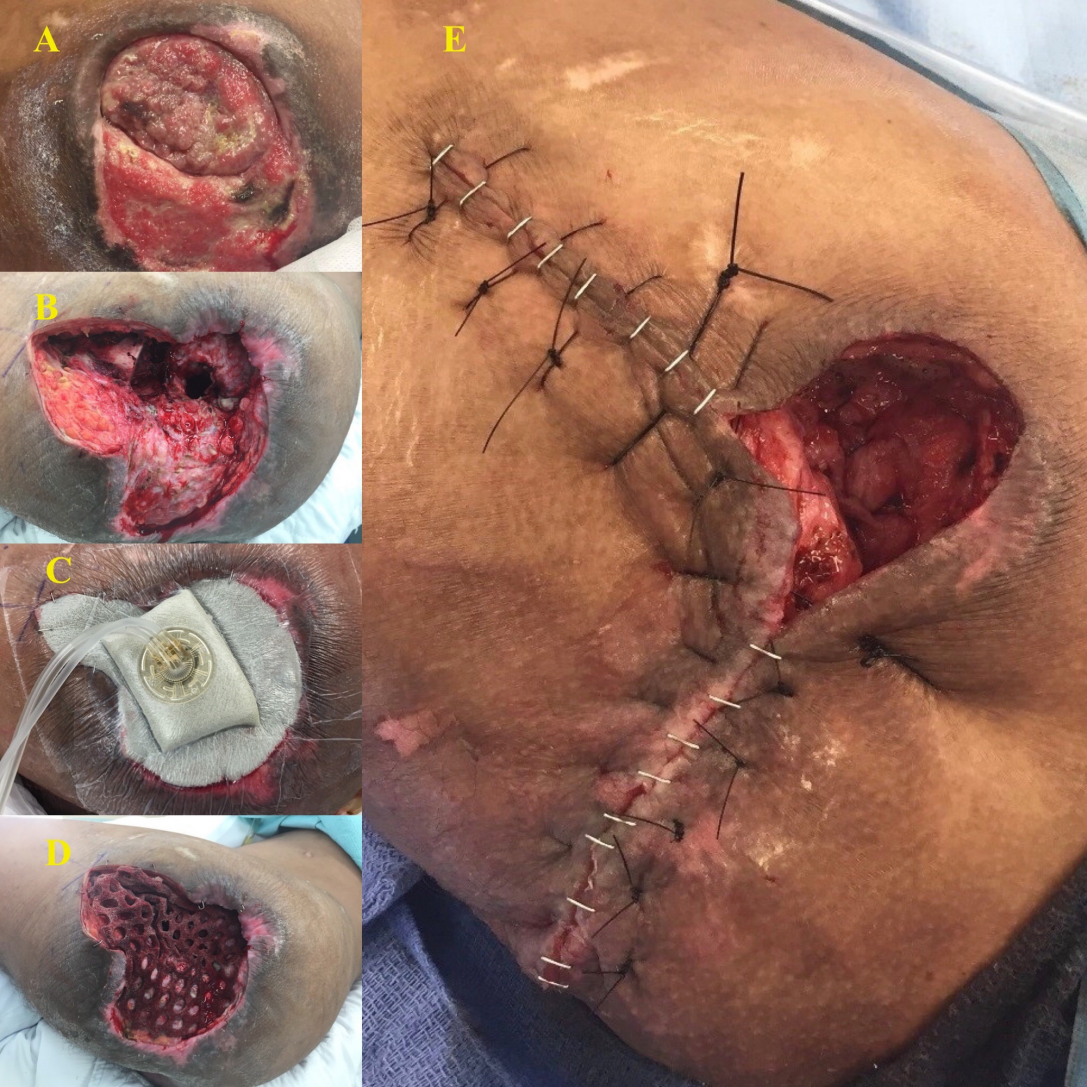
Patient 7: Left Hip A: Wound; B: Post-operative wound; C: Negative pressure wound therapy with instillation and dwell; D: Wound after treatment with instill E: Delayed primary closure

Due to the success of the right Girdlestone procedure, the patient underwent a left Girdlestone approximately three months later. Like the right side, he had developed a chronic ulcer over the left greater trochanter with subsequent femoral head osteomyelitis. Intraoperative findings were also similar, with a necrotic femoral head and resultant cultures growing no organisms, though previous cultures grew Pseudomonas. The wound was dressed with a Cleanse Choice NPWTi-d (see Figure 6 below). Three days later, he underwent a partial delayed primary closure over closed suction drains with the placement of a negative pressure device over the incision. He was discharged eight days after the initial procedure on doxycycline and trimethoprim-sulfamethoxazole and was not readmitted in the first 30 days after discharge.

The patient was readmitted at 60 days with concern for the protrusion of his left distal femur into the ongoing wound bed and was taken to the operating room for excisional debridement and bone biopsy. The biopsy was negative for invasive osteomyelitis. In addition, at month four, he presented with a stage IV ulceration of his sacrum. Adequate offloading, wound care and nutritional support, and intravenous antibiotics were not able to be achieved in the postoperative care of this patient due to numerous factors. He was discharged in this state five days later on trimethoprim-sulfamethoxazole with the intent to heal by secondary intention and has since re-presented with progressive malnutrition and dry gangrene of the toes of his right leg. He has refused ongoing medical care. He has not required ongoing treatment for the infection in either hip and his surgical wounds continued to decrease in size.

Patient 8

This was a 59-year-old male with a history of paraplegia with progressive worsening of his multiple sacral and ischial stage IV pressure ulcers, resulting in chronic osteomyelitis and dislocation of his right femoral head. He presented for a Girdlestone procedure on the right side. Intraoperative findings were significant for a necrotic femoral head. Cultures were sterile at this time, but previous cultures of the same wound grew Staphylococcus capitis, Candida albicans, Acinetobacter calcoaceticus-baumannii complex, and mixed flora. The wound was dressed with a Cleanse Choice NPWTi-d initially in addition to the application of a collagen, cellulose, and silver matrix. Instillation was started with normal saline on postoperative day one, once hemostasis was assured. Four days later, he underwent a delayed primary closure over closed suction drains with the placement of a negative pressure device over the wound incision, which was completely closed. His drain was dislodged prematurely postoperatively and he developed a partial dehiscence of his wound in the area of his previously open ischial pressure ulcer. He was discharged 17 days after the first procedure on ceftriaxone and vancomycin, with gauze dressing changes for the area of dehiscence. He was unable to continue negative pressure wound therapy. He was not readmitted in the first 30 days after discharge.

During his first two months postoperatively, he was lost to follow-up by the infectious disease and surgical clinics and presented then with a clean and healing wound bed. He was readmitted three months postoperatively with a concern for progressive osteomyelitis of his sacrum, which was negative on biopsy and was found instead to have polymicrobial urosepsis. At his six-month follow-up, he was found to have a continued decrease in his wound size without complete healing, however, there was no recurrence of his invasive osteomyelitis.

Patient 9

This was a 52-year-old male with a history of a severe peripheral vascular disease, right hip disarticulation, and paraplegia, with a stage IV pressure ulcer of the left greater trochanter with resultant osteomyelitis. He underwent an attempt at limited resection of his greater trochanter in a two-stage procedure but developed an invasive infection of his hip and acetabulum with ongoing wound drainage and dehiscence. Thus, he was counseled and planned for definitive therapy with a Girdlestone procedure. Intraoperative findings included a grossly necrotic femoral head with a large open wound over the greater trochanter. Intraoperative cultures grew Staphylococcus epidermidis, which had been isolated at the prior operation. The wound was dressed with a Cleanse Choice NPWTi-d. Three days later, he underwent a delayed primary closure over closed suction drains with the placement of a Prevena negative pressure device over the incision. He was discharged seven days after the first procedure on ceftriaxone and vancomycin. He was not readmitted in the first 30 days after discharge.

**Figure 7:**
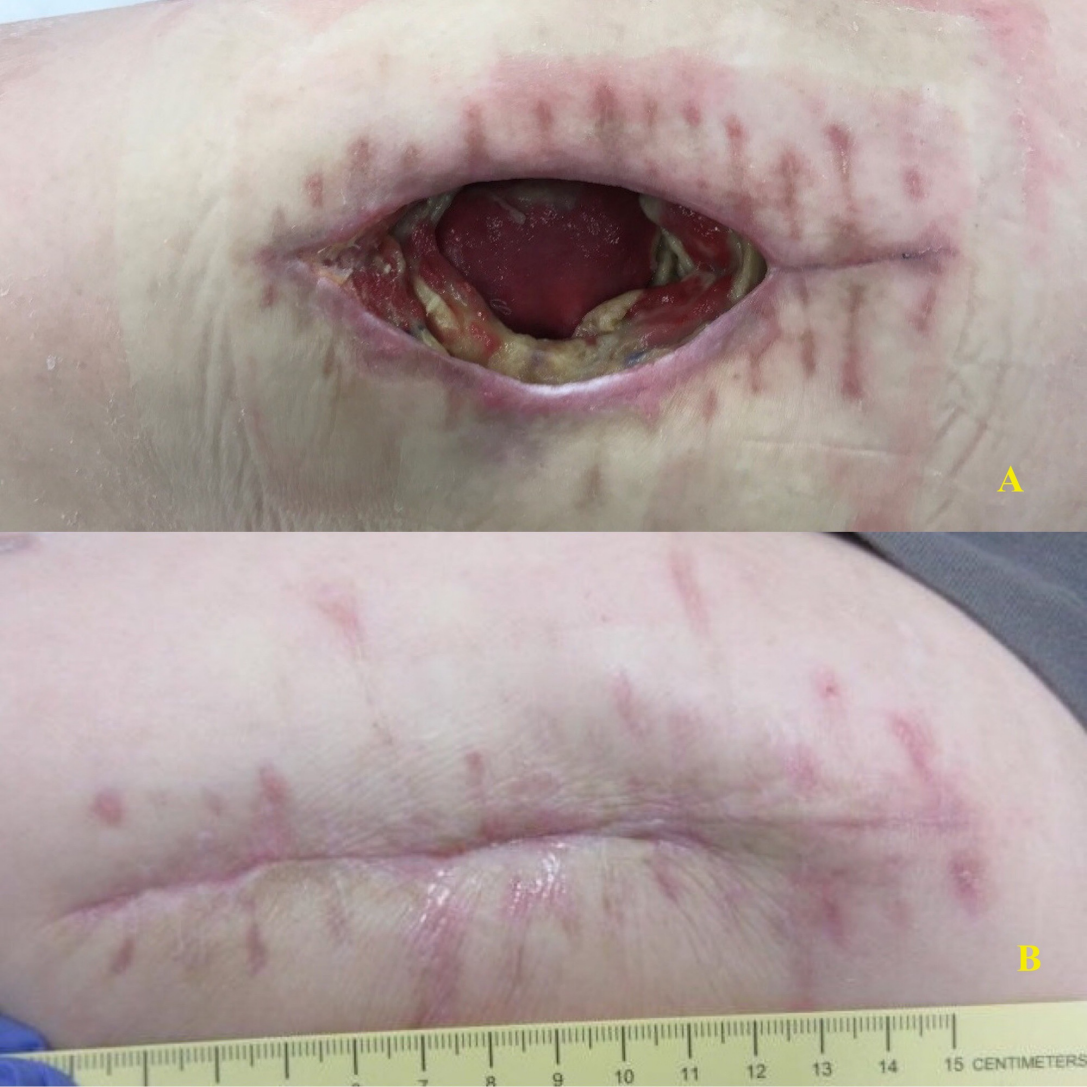
Patient 9 A: Wound prior to Girdlestone; B: Healed wound at follow-up

While his incision has healed completely (see Figure 7 below), he has persistent low volume drainage via a surgical drain at five months postoperatively but no clear radiographic or clinical evidence of recurrent osteomyelitis.

Patient 10

**Figure 8:**
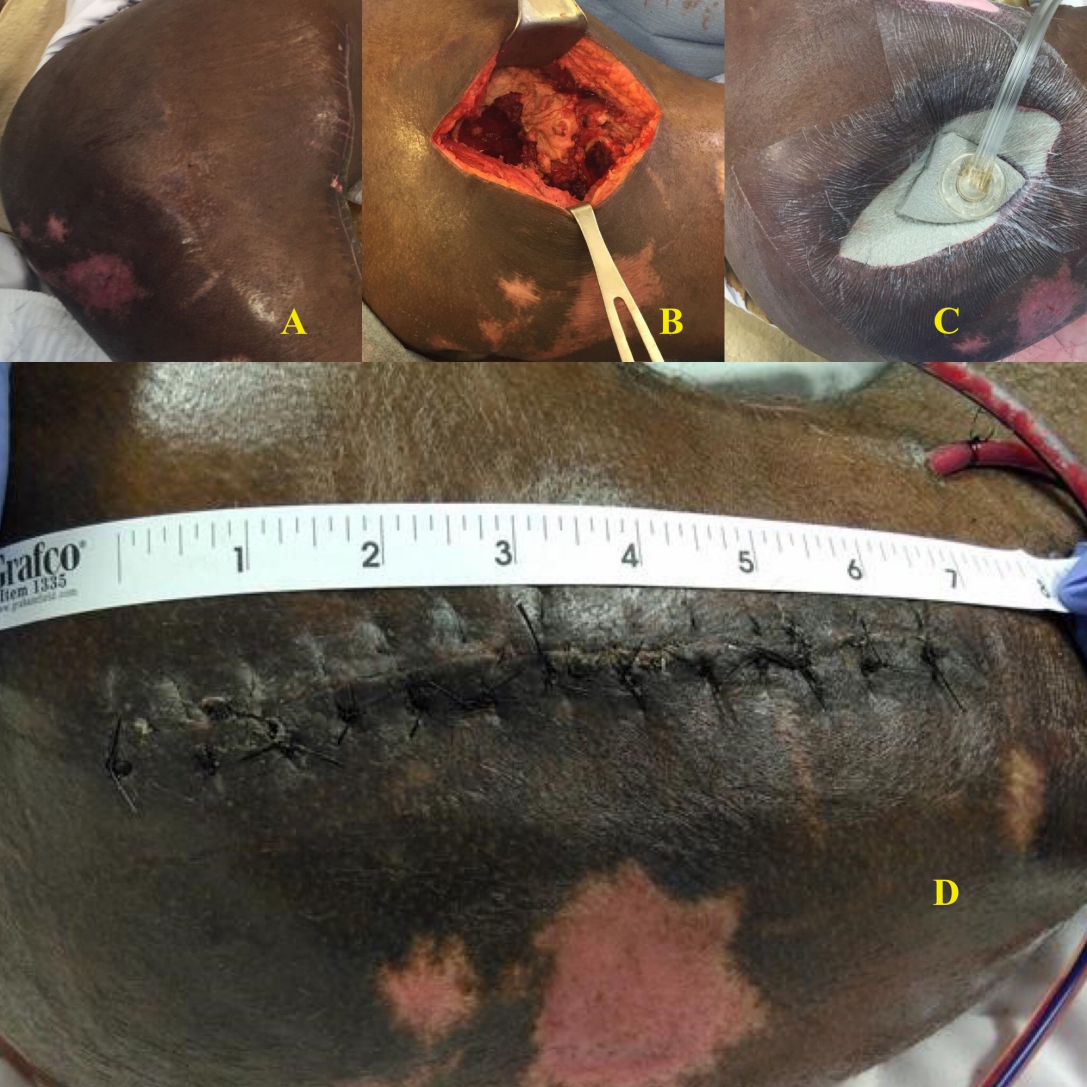
Patient 10 A: View of hip prior to Girdlestone; B: Intraoperative view of grossly purulent tissue; C: Instillation therapy; D: Delayed primary closure

This was a 21-year-old male with a history of paraplegia who had recurrent septic arthritis of the right hip due to MRSA, which failed to resolve after open drainage was performed at an outside hospital. He presented with sepsis, MRSA bacteremia, and acute chronic osteomyelitis in the femoral head, with an extensive invasive soft tissue infection involving the entire gluteus, posterior compartment, and hip. He underwent an emergent Girdlestone procedure. Intraoperative findings were significant for a completely necrotic femoral head surrounded by fluid, with extensive purulence throughout the acetabulum and surrounding soft tissue (see Figure 8 below). Cultures were significant for MRSA. The wound was dressed with a negative pressure device without instillation and dwell initially and then transitioned to NPWTi-d with 75 mL instillation of ¼ strength Dakin's solution for a 10-minute dwell time at 3.5-hour intervals once his hemoglobin stabilized. Two days later, he underwent a further debridement of the right Girdlestone site and ulcer, with a changing of the negative pressure device sponge. Due to the degree of invasive infection, a third operation was performed to obtain source control with a reinitiation of instillation therapy. Four days later, a total of 13 days after the initial procedure, he underwent delayed primary closure over closed suction drains with the placement of a negative pressure device over the incision. He was discharged 20 days after the initial procedure on fluconazole, cefepime, metronidazole, and vancomycin.

He was not readmitted in the first 30 days after discharge, but he was unable to comply with ongoing offloading and wound care. Despite this, his lateral incision healed well postoperatively but he presented approximately eight weeks after the initial presentation with a progression of the ischial pressure ulcers and septic arthritis on his contralateral hip with acute dislocation of his femoral head for which he underwent a Girdlestone procedure as well.

## Discussion

Chronic osteomyelitis is a severe infectious process that can be difficult to treat. Historically, the Girdlestone procedure was known as an effective approach to treating septic arthritis in the hip; however, it has lost favor due to the morbidity of loss of ambulation and a prolonged open wound. In this case series, 11 cases of invasive osteomyelitis in non-ambulatory patients were managed with Girdlestone pseudoarthroplasty. Overall, this case series suggests that the Girdlestone procedure combined with the postoperative administration of instillation therapy and delayed primary closure represent a novel technique for the treatment of refractory invasive infection with favorable wound healing and even primary closure. Oheim and colleagues studied Girdlestone pseudoarthroplasty in the context of a postoperative infection or hematologic spread of infection to the hip [10]; the Girdlestone controlled 96% of infections in that study as compared to 100% in our series. In a more general study, Rennert and colleagues surveyed the electronic medical record for cases of osteomyelitis related to stage IV pressure ulcers and found that the surgical debridement of bone, which operates under a similar principle as the Girdlestone, caused a decrease in the amount of infected tissue in 76% of the patients [17]. Our series here showed excellent results in 100% of patients, with no recurrence in the follow-up period and no deaths [18]. The most common complication noted in our series was the worsening of another pressure ulcer. Each of these findings presented greater than 30 days postoperatively and were not a direct complication of the surgical procedure but, rather, a reflection of the patient’s nutritional status, co-morbidities, and access to adequate wound care and offloading.

In order to close the large tissue defect left by the resection of the femoral head, previous works focused on the use of a large muscle flap, usually the vastus lateralis, which is a major and technically challenging surgery involving tedious dissection and greater anesthesia and surgical risks. These flaps also fail, as 31%-43% have a recurrence of the ulceration over the treated hip with wound breakdown [19-20]. In our series, the application of NPWTi-d and delayed primary closure with topical negative pressure therapy results in no recurrence of ulceration over the treated hip and no breakdown of the wound. Rather than healing only by secondary intention, NPWTi-d provided an adjunct to the treatment of infection and, combined with incisional negative pressure therapy, facilitated delayed partial and complete primary closure. The existence of a preoperative pressure ulcer of the greater trochanter was the single most important factor in patients being able to achieve primary wound closure, as all four patients with no previous ulcer were closed primarily and had no wound complications and three with a prior ulcer achieved complete or near complete healing at the time of submission of this study.

The limitations of this series include the small number of patients included and the narrow indication used. In addition, patients presented with a wide variation of pre-existing nutritional status, co-morbidities, and pre-operative pressure ulcers. Finally, follow-up was only taken through a minimum of one month; if further follow-up was available, it was discussed, but this was not true for some patients.

## Conclusions

Girdlestone pseudoarthroplasty is a radical but effective technique in eliminating invasive osteomyelitis of the femoral head and acetabulum with a recurrence rate of 0% in this 10-patient series, encompassing 11 Girdlestone procedures. When combined with NPWTi-d, patients who undergo Girdlestone pseudoarthroplasty can be closed primarily in a delayed fashion, rather than healing by secondary intention, with excellent results. Further study of the effectiveness of Girdlestone pseudoarthroplasty in the treatment of invasive osteomyelitis is warranted in the form of prospective evaluation.
